# NSAID overuse leading to cardiac aneurysm: Unveiling a missed diagnosis of Behçet’s disease: A case report

**DOI:** 10.1097/MD.0000000000044111

**Published:** 2025-08-22

**Authors:** Farah Jaafar Mahdi, Ahmed Dheyaa Al-Obaidi, Ghadeer Mohammed, Aya Ahmed Shimal, Marafi Jammaa Ahmed, Hashim Talib Hashim, Ibrahim Gowaily, Hasan Al-Obaidi

**Affiliations:** a Rheumatology Unit, Department of Medicine, College of Medicine, University of Mustansiriyah, Baghdad, Iraq; b Department of Medicine, University of Baghdad, College of Medicine, Baghdad, Iraq; c Department of Medicine, University of Kufa College of Medicine, Najaf, Iraq; d Department of Medicine, College of Medicine, University of Baghdad, Baghdad, Iraq; e Faculty of Medicine, Bahri University, Khartoum, Sudan; f Vascular Surgery Department, Oregon Health and Science University, Portland, OR; g Department of Medicine, University of Warith Al-Anbiyaa, College of Medicine, Karbala, Iraq; h Faculty of Medicine, Tanta University, Tanta, Egypt; i Department of Medicine, Jamaica Hospital Medical Center, New York City, NY.

**Keywords:** Behçet’s disease, immunosuppression, left ventricular aneurysm, multimodal therapy, NSAID, thromboembolism

## Abstract

**Rationale:**

Behçet disease (BD) is a chronic inflammatory vasculitis that primarily affects young males and is associated with severe vascular complications. Prolonged nonsteroidal anti-inflammatory drug (NSAID) use may mask early inflammatory signs, delaying diagnosis and appropriate treatment.

**Patient concerns:**

A 28-year-old pharmacist experienced syncope, chest pain, and leg swelling after prolonged NSAID use.

**Diagnoses:**

Workup revealed a left ventricular aneurysm, deep vein thrombosis, and multiple arterial occlusions, leading to the diagnosis of BD.

**Interventions:**

The patient received therapeutic anticoagulation, high-dose corticosteroids, cyclophosphamide, colchicine, and later azathioprine as maintenance. Surgical interventions included left ventricular aneurysmectomy and femoropopliteal bypass.

**Outcomes:**

The patient showed dramatic clinical improvement with resolution of oral ulcers, normalization of cardiac function, regression of thrombus, and improvement from New York Heart Association class IV to II at 1-month follow-up. No new thrombotic or vascular events occurred during subsequent years under maintenance immunosuppression.

**Lessons:**

This case underscores the life-threatening consequences of undiagnosed BD and highlights how NSAID overuse can obscure early inflammatory symptoms, delay immunosuppressive therapy, and lead to severe cardiac complications. Early recognition, cautious NSAID use, and a multidisciplinary approach are essential in managing BD-related vascular disease.

## 1. Introduction

Behçet’s disease (BD) is a chronic, multisystemic vasculitis first described in 1937 by Turkish dermatologist Hulusi Behçet. It is characterized by immune-mediated inflammation affecting the venous and arterial systems, leading to recurrent oral and genital ulcers, cutaneous lesions, uveitis, and systemic vasculitis. The pathogenesis of BD involves dysregulated immune responses induced by genetic and environmental factors, resulting in endothelial dysfunction, neutrophilic vascular infiltration, and hypercoagulability.^[1]^ Although cardiac involvement is rare, it can lead to pericarditis, endomyocardial fibrosis, or myocardial infarction (MI).^[2]^ Arterial manifestations, occurring in 1.5% to 2.3% of cases, are clinically significant due to their tendency to form aneurysms rather than thromboses, with postoperative complications such as anastomotic pseudoaneurysms substantially increasing morbidity and mortality. It primarily affects young adults, demonstrating a marked male predominance. Males experience more severe vascular involvement, poorer prognosis, and higher mortality rates compared to females^[3]^ The diagnosis adheres to the International Criteria, which assigns weighted scores to clinical features: oral aphthosis (2 points), genital ulcers,^[2]^ ocular lesions,^[2]^ and skin, vascular, or neurological manifestations or a positive pathergy test (1 each); a score ≥ 4 confirms the diagnosis.^[4]^

Nonsteroidal anti-inflammatory drugs (NSAIDs) are commonly used to manage musculoskeletal symptoms, their prolonged unsupervised use poses diagnostic challenges. By suppressing systemic inflammatory markers, NSAIDs can obscure disease progression and delay the timely identification of severe organ involvement and may lead to delays in diagnosis and proper therapy.^[5,6]^

This case describes a 28-year-old man with a complicated Behçet’s disease. His long-term NSAID use hid signs of inflammation, delaying diagnosis of Behçet’s disease complicated by a life-threatening heart complication documented in fewer than 20 cases worldwide (left ventricular [LV] aneurysm). This case highlights the critical need to reevaluate young males with mucocutaneous ulcers and thrombosis unresponsive to conventional therapies.

## 2. Case presentation

A previously healthy 28-year-old male pharmacist from Iraq presented to the emergency department after collapsing at work, following a 1-week history of progressive dyspnea and severe central crushing chest pain, followed by acute right lower extremity swelling. On examination, he had 3 well-defined oral ulcers (1 cm diameter, necrotic base, erythematous borders). Vitally, he had tachycardia (125 bpm), low-grade fever (38°C axillary) with elevated jugular venous pressure. Precordial examination revealed a displaced apex beat, gallop rhythm. Lowe limb examination revealed erythema, warmth, pitting edema and an increase of 5 cm in circumference in right leg compared to the contralateral limb with a preserved dorsalis pedis and posterior tibial pulses.

Two months prior to his admission, the patient experienced recurrent oral and genital ulcers. Followed by the onset of symmetric polyarthralgia affecting the small joints of his hands and feet, accompanied by 30-minute morning stiffness. In an attempt to manage his symptoms, he self-prescribed prolonged high-dose NSAID use (ibuprofen 400 mg, 3–4 times daily), with associated gastric upset. This excessive NSAID use likely masked early ischemic symptoms, contributing to diagnostic delay and progressive myocardial damage.

**Figure 1.**
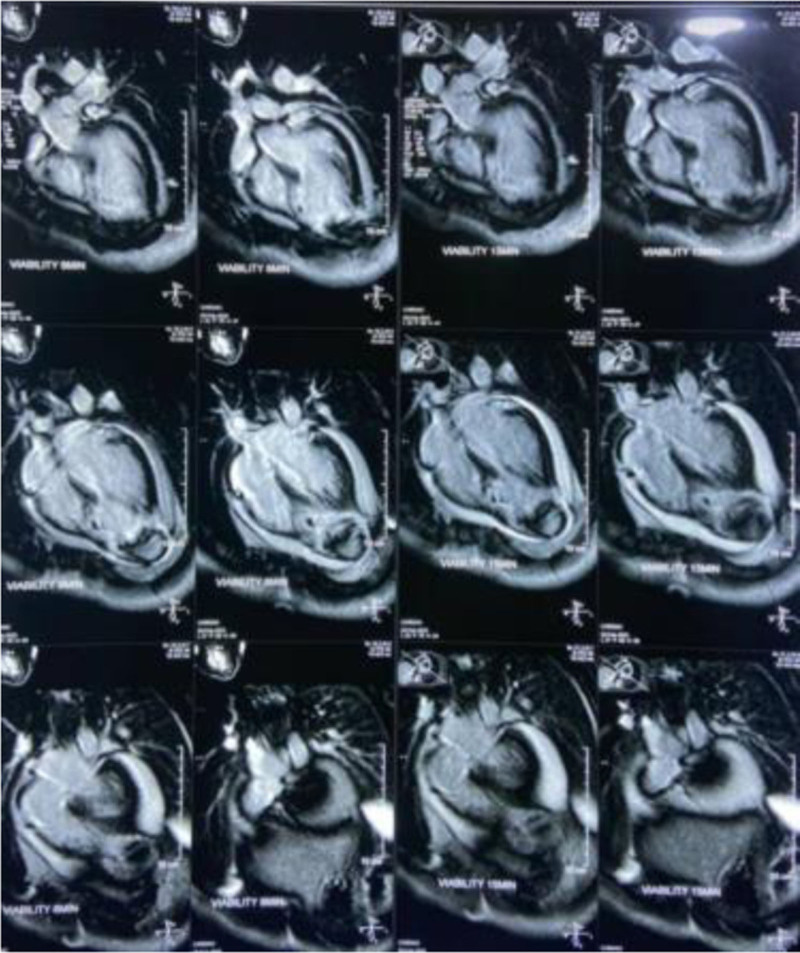
The cardiac MRI series showing wall thinning in the anterolateral region. And hypointense areas indicative of mural thrombus formation. MRI = magnetic resonance imaging.

ECG showed ST-segment elevation with pathological Q waves in the anterolateral leads, while laboratory tests revealed elevated high-sensitivity troponin (0.022 ng/mL), along with increased C-reactive protein and erythrocyte sedimentation rate levels. This confirmed the diagnosis of ST-elevation MI with the subsequent start of anti-ischemia measures and thrombolytics. Follow-up ECG showed persistent ST-elevation over the next few days. Transthoracic echocardiography revealed severe LV dysfunction (ejection fraction 33%), apical hypokinesis, and moderate pericardial effusion. Cardiac MRI confirmed a LV aneurysm with myocardial wall thinning in the anterolateral region, mural thrombus, and delayed enhancement, indicating extensive myocardial necrosis and fibrosis (see Fig. 1). Doppler ultrasound identified absent flow caused by thrombosis in the right superficial femoral, popliteal, and posterior tibial veins. CT angiography demonstrated total occlusion of the right external iliac and common femoral arteries.

Behçet’s disease (BD) was diagnosed. The diagnosis of Behçet’s disease was established using the International criteria for Behçet’s disease (ICBD). The patient presented with recurrent oral ulcers (2 points), genital ulcers reported 2 months prior to admission (2 points), and significant vascular involvement including deep vein thrombosis and arterial occlusion (1 point), yielding a total score of 5, meeting the diagnostic threshold of ≥4. These features, combined with elevated inflammatory markers and absence of conventional cardiovascular risk factors, strongly supported BD as the underlying etiology. The pattern of multiple vascular thrombosis in a young adult, along with mucocutaneous involvement and after thorough exclusion of alternative diagnoses, further reinforced the diagnosis. Immunosuppressive therapy was initiated with pulse methylprednisolone (1g/day for 3 days) with subsequent prednisolone 1 mg/kg per day and 6 doses of monthly cyclophosphamide, warfarin, and daily oral colchicine. The patient improved dramatically with resolution of chest pain, stabilization of cardiac function, and thrombus regression. Given the persistence of lower limb arterial occlusion, femoropopliteal bypass surgery was performed. He was kept on azathioprine as a maintenance dose after completion of his cyclophosphamide doses. At 1-month follow-up after initiating immunosuppressive therapy, the patient reported complete resolution of oral ulcers and showed marked clinical improvement. Cardiac function normalized with restoration of a normal ejection fraction, and his symptoms improved from New York Heart Association functional class IV to class II. No new thrombotic or vascular complications were observed during follow-up in the subsequent years, indicating a stable disease course under maintenance immunosuppression.

## 3. Discussion

Behçet’s disease is a rare, multisystem vasculitis that has a complex and heterogeneous presentation. This report is of a rare presentation of Behçet’s disease (BD) involving widespread vasculitis of the arterial and venous circulations that later leads to MI complicated by LV aneurysm development. The patient was identified as having a classic presentation of BD, with recurrent oro-genital ulcers, DVT, and arterial thrombosis that, in the setting of systemic inflammation, all pointed significantly in the direction of BD being the cause rather than a primary atherosclerotic MI.

The differential diagnosis of BD is enormously broad, it may mimic several autoimmune and autoinflammatory diseases. Common mimickers include systemic lupus erythematosus, which shares mucocutaneous and vascular features but differs by the presence of serologic markers like ANA and dsDNA. Crohn disease can resemble BD in gastrointestinal and oral ulcers, but Crohn shows characteristic ileocecal ulcers and granulomas on endoscopy. Spondyloarthritis may present with arthritis and uveitis, similar to BD, but typically lacks genital ulcers and erythema nodosum-like lesions. Multiple sclerosis can mimic neuro-BD but differs in MRI findings and CSF profile. In contrast to infectious ulcers (e.g., HSV), BD ulcers are sterile and recurrent.^[7–9]^ Expert clinical judgment and longitudinal follow-up remain crucial in accurately diagnosing BD and ruling out its mimickers.

Cardiac manifestations in Behçet’s disease may come in the form of pericarditis, myocarditis, endocarditis, intracardiac thrombus, and occasionally coronary artery involvement with MI.^[1–3]^ These complications are ostensibly driven by chronic inflammation, oxidative stress, and endothelial dysfunction, which result in a prothrombotic state. Vasculitis-induced damage further contributes to coronary aneurysms, thrombosis, and MI, with severity linked to disease activity.^[4]^

In our case, the patient presented with extensive thrombotic disease involving multiple vascular territories, including the iliac and common femoral arteries, as well as the deep veins of the right lower extremity. This is in agreement with recent reports that outlined the diagnostic pitfalls of intracardiac thrombosis in Behçet’s disease (BD), where Behçet’s presents with intracardiac thrombi, hemoptysis, and dyspnea with the common association of pulmonary embolism or aneurysms.^[10–12]^ These findings emphasize the need to consider BD in the differential diagnosis of intracardiac masses, particularly in young adults.

Further supporting this, recent research utilizing more advanced imaging technologies has recognized mild cardiac abnormalities in BD patients. Notably, a cardiac MRI study found that 83% of BD patients with no history of cardiac disease had at least 1 abnormality, such as myocardial edema, valvular regurgitation, or coronary artery aneurysms.^[13]^

Histological findings in BD frequently demonstrate neutrophilic infiltration and vascular wall destruction, contributing to aneurysm formation.^[14]^ The underappreciated danger of NSAID overuse in systemic vasculitis like BD cannot be overstated. Our patient had a LV aneurysm that more likely developed due to multiple unrecognized MIs, exacerbated by the inappropriate use of NSAIDs. The self-medication of high-dose ibuprofen before admission, while providing symptomatic relief, has masked ischemic pain, delayed diagnosis, and contributed to progressive myocardial damage. Therefore, NSAID use in patients with undiagnosed systemic inflammation should prompt caution, especially in settings where autoimmune etiologies may underlie vague symptoms.

For instance, there is emerging evidence that suggests that NSAIDs may contribute to aneurysm formation by impairing collagen remodeling and increasing the risk of myocardial rupture post-MI. Though the exact mechanism is unclear, NSAID-induced vascular changes may weaken the myocardium, promoting aneurysm development.^[15–17]^ This risk potentially calls for attention when prescribing NSAIDs during acute cardiovascular events.

Initial treatment of Behçet’s disease (BD) typically involves glucocorticoids and immunosuppressive agents like azathioprine and cyclophosphamide. The use of anticoagulants in BD patients with vascular involvement remains controversial, with venous and arterial involvement generally treated with immunosuppressants rather than anticoagulants.^[18]^

This case emphasizes the management challenge in vascular BD, where immunosuppression remains the primary treatment, but surgical intervention is necessary for complex structural complications.

Ultimately, this case underscores the importance of considering BD as a differential diagnosis for thrombosis and MI, particularly in young patients without typical cardiovascular risk factors. further, it highlights concerns regarding NSAID abuse, which can mimic ischemic symptoms and contribute to aneurysm formation. Early recognition and targeted therapy are crucial to preventing severe complications in BD-related vascular disease.

## 4. Conclusion

The presented case reports the atypical presentation of LV aneurysm and thrombus in a young man with Behçet’s disease and NSAID abuse. It highlights the need for stricter NSAID monitoring in patients with unexplained vascular or mucocutaneous symptoms, as NSAIDs can mask inflammation, delay diagnosis, and worsen vascular damage in systemic diseases like Behçet’s. Clear guidelines on NSAID use in suspected inflammatory disorders are urgently needed. Association of the manifestations of Behçet’s disease with acute MI presents a diagnostic challenge. This case emphasizes the importance of individualized treatment and follow-up in patients with Behçet’s disease to prevent life-threatening complications. Maximizing management, especially inflammatory activity and the use of NSAIDs, needs further studies.

## Author contributions

**Conceptualization:** Farah Jaafar Mahdi, Hasan Al-Obaidi.

**Data curation:** Ghadeer Mohammed, Marafi Jammaa Ahmed, Hashim Talib Hashim, Ibrahim Gowaily.

**Investigation:** Ahmed Dheyaa Al-Obaidi.

**Methodology:** Farah Jaafar Mahdi, Ahmed Dheyaa Al-Obaidi, Aya Ahmed Shimal, Hashim Talib Hashim, Ibrahim Gowaily.

**Project administration:** Aya Ahmed Shimal.

**Resources:** Ghadeer Mohammed, Hashim Talib Hashim.

**Supervision:** Hasan Al-Obaidi.

**Writing – original draft:** Farah Jaafar Mahdi, Ahmed Dheyaa Al-Obaidi, Ghadeer Mohammed, Aya Ahmed Shimal, Marafi Jammaa Ahmed, Ibrahim Gowaily.

**Writing – review & editing:** Marafi Jammaa Ahmed, Hasan Al-Obaidi.
